# Targeting transthyretin deposition in lumbar spinal stenosis: a mechanistic rationale for tafamidis

**DOI:** 10.3389/fsurg.2026.1816506

**Published:** 2026-05-07

**Authors:** Iván Z. González, Giovanni Ureña

**Affiliations:** Department of Neurosurgery, Centers for Diagnostics, Advanced Medicine, and Telemedicine (CEDIMAT), Santo Domingo, Dominican Republic

**Keywords:** kinetic stabilization, ligamentum flavum hypertrophy, lumbar spinal stenosis, tafamidis, transthyretin amyloidosis (ATTR)

## 1 Introduction

Transthyretin (TTR) is a 127-amino acid protein that forms a homotetramer, whose main function is to transport thyroxine and retinol-binding protein-retinol (vitamin A) complex (1). Misfolding of TTR leads to transthyretin amyloidosis (ATTR), which is associated with cardiomyopathy (ATTR-CM) and polyneuropathy (ATTR-PN). There are two types of ATTR: a wild-type (previously called senile; ATTRwt) and a variant-type (genetic variation; ATTRvt) (2). More than 150 pathogenic TTR proteins have been identified in ATTRvt, the most common being Val122Ile (TTRVal122Ile), and the one with the most penetrance, TTRVal30Met (3). Furthermore, in a recent survey, TTRVal30Met was responsible for more than half of amyloidosis cases, followed by ATTRwt and TTRVal122Ile, and it was noted that ATTRwt was a more common cause of symptomatic amyloidosis in North America (59.5%) (4).

Under these conditions, an association between ATTR and lumbar spinal stenosis (LSS) has been found, due to the presence of TTR deposition in the ligamentum flavum (LF) in patients undergoing LSS decompression alongside a diagnosis of ATTR-CM, which may contribute to LF thickening and canal narrowing. There is no exact epidemiologic data about LSS, but a recent JAMA review highlighted its prevalence of 11%, and it is estimated to afflict more than 200,000 individuals in the US, and it is considered the most common indication for spinal surgery in patients over 65 years of age (5–8) The first study hypothesizing about the role of ATTR in lumbar spinal stenosis found 21 out of 26 lumbar LF specimens with amyloid (9). Another study analyzed the presence of TTR in LF in patients aged over 50 years old undergoing lumbar stenosis (LS) surgery, and found a prevalence of 37% of TTR amyloid deposits in vertebral ligament tissue, but a more recent study showed an incidence of 48% of these deposits (10, 11). In addition, an article highlighted a correlation of 0.54 between ATTR in LF and its thickness (12). There has been speculation that ligamentous tissue may be the primary place for ATTR fibrillogenesis by yet unknown mechanisms (10). Although the contribution of ATTR deposition to reoperation risk remains unproven, lumbar decompression carries an overall reoperation rate of up to 22%. This highlights the need to explore adjunctive strategies that may address potential biological contributors to disease progression (13).

In recent years, new medications for ATTR have been developed. One of these is the TTR stabilizer tafamidis meglumine (Vyndaqel), which is currently used for the management of both ATTR-CM (since 2019, after the ATTR-ACT trial in 2018) and ATTR-PN (since 2011, in Europe), independent of the type of TTR (wild or variant) (14–16) It works by preventing the degradation of TTR tetramers into monomers by uniting with thyroxine-binding sites via kinetic stabilization (17). There are other less studied medications, such as TTR silencers (patisiran, inotersen), and the NSAID-based counterpart of tafamidis, diflunisal, both sharing the same mechanism of action. At least for ATTR-CM, no current clinical data justify the use of combination therapy with these drugs, although an argument that it could enhance TTR stabilization and improve outcomes is under debate (18).

The inquiries of the current article seek to illustrate the possible role that tafamidis may exert in lumbar spinal stenosis. Also, defining whether the drug has a radiologically detectable reduction in ligamentum flavum thickness is pertinent; until now, this has been hypothesized but not demonstrated. This may represent one of the first mechanism-driven pharmacologic approaches targeting the biological substrate of degenerative lumbar spinal stenosis. The repurposing of a previously investigated medication, which has been shown to have an adequate safety profile with treatment-emergent adverse event (TEAE) incidence similar to placebo (independent of the tafamidis dose studied), can have a pivotal role in this patient population (19). We propose that TTR stabilization may represent a disease-modifying adjunct in a biologically defined subset of lumbar spinal stenosis patients.

### 1.1 ATTR deposition in LF

The etiology of LSS is multifactorial, with contributors such as LF hypertrophy (LFH), or thickening, degenerative disc disease, and facet joint hypertrophy being the main culprits (20). Chronic inflammation due to angiopoietin-like protein 2 (Angptl2) and interleukin-6 (IL-6) is likely responsible for the reduced elasticity and fibrosis of LF, as well as genetic predisposition (20). The pathogenesis of LFH is not well understood, but decreased elasticity due to collagen and ATTRwt deposition is reported (21). The presence of the latter is associated with a higher burden of LFH compared to its absence, elevating the clinical relevance and therapeutic options (22). A study reported 88.4% of amyloid deposition (total) in LF in males and females of 50 years of age and older, of which 37.2% was ATTR (10). Moreover, the Cardiac Amyloidosis in Spinal Stenosis (CASS) trial found that 59% of the 82 participants undergoing spinal stenosis surgery had a high prevalence of ATTR amyloid deposits in LF biopsy (23).

### 1.2 LSS as a sentinel event to ATTR

Several studies have highlighted an association between ATTR-CM and LSS (24). It is known that LSS can precede the diagnosis of ATTR-CM on average by 5 to 15 years, in up to 40% of cases (23). Also, more than half of spinal stenosis patients have screening criteria for cardiac amyloidosis, given they share the same age group (25).

To date, there is no indication for routine LF biopsy in order to detect ATTR earlier, and thus, a preventive approach to ATTR-CM. A clinical trial is currently being executed, with the main objective of evaluating the presence of amyloidosis in patients undergoing surgery for non-congenital spinal stenosis and/or spondyloarthropathy (23). The way toward LF biopsy for a specific subset of vulnerable patients at risk of developing ATTR-CM can be a leap forward in both diagnostics and prompt treatment. Further, although there is no guideline or validated indication for specific cardiac biomarkers in suspected ATTR-CM, several markers are significantly correlated with the disease, and thus should be thoroughly examined in cases of LSS (26, 27). For these reasons, it would be ideal to have a screening protocol in patients undergoing decompressive spine surgery who meet the following criteria: over 50 years of age, bilateral carpal/cubital tunnel syndrome history, biceps tendon rupture, and elevated NT-proBNP without a clear cause (28).

### 1.3 Mechanistic plausibility of TTR stabilization in LSS

The management of LSS ranges from non-surgical (e.g., drugs such as analgesics, prostaglandins, and vitamin B1; physiotherapy; epidural injections) to surgical (i. e., decompression) (7). In a biologically defined subset of patients, LSS may be a microscopic, molecular problem, which is later transformed into a macroscopic, surgical condition. TTR misfolding is a systemic process that may continue after decompression and potentially involve spinal levels not previously affected.

Tafamidis is a small molecule with high plasma protein binding that stabilizes circulating TTR tetramers (16, 17). Although direct evidence of tafamidis penetration into ligamentous tissue is lacking, TTR itself is a circulating protein that deposits systemically, suggesting that stabilization at the plasma level may reduce downstream tissue deposition. This indirect mechanism provides a biologically plausible rationale for evaluating tafamidis in LF amyloid disease, although tissue-specific pharmacokinetics remain to be established.

### 1.4 Clinical trial design considerations

There is no proof that TTR deposition drives LFH, nor that tafamidis can reach avascular ligamentous tissue such as LF, or that TTR stabilization may influence local deposition kinetics. Consequently, a phase IIa, exploratory trial might be adequate, delimiting tissue, imaging, and functional endpoints, as well as testing the aforementioned biological plausibility (29).

The trial can include the following patients: age ≥ 60, clinical and radiological (MRI) LSS, LF thickness ≥ 4 mm, histologic confirmation of ATTR deposition in LF from prior decompression surgery or intraoperative biopsy at time of index decompression (30). To protect internal validity, patient safety and biological clarity, exclusion criteria may be: non-degenerative spinal pathology (these can cause stenosis mechanically, independent of LF hypertrophy), including congenital spinal canal stenosis, high-grade spondylolisthesis (grade III-IV), severe scoliosis (>25° Cobb angle), active spinal infection, active spinal malignancy, acute traumatic spinal injury; prior decompression at index level, due to scar tissue altering spinal biomechanics; non-TTR amyloidosis (confirmed AL amyloidosis) and confirmed advanced ATTR-CM/PN; and life expectancy < 2 years due to non-cardiac disease. Advanced ATTR-CM will be defined according to the Gillmore staging system as Stage III disease (NT-proBNP >3,000 ng/L and eGFR <45 mL/min/1.73 m^2^), given its association with poor prognosis and limited expected benefit from disease-modifying therapy (31). Advanced ATTR-PN will be defined as stage 3 polyneuropathy, characterized by wheelchair dependence or severe functional impairment (32). These patients are excluded to avoid confounding due to systemic disease burden and because tafamidis is already indicated in these populations.

All participants will undergo TTR gene sequencing to differentiate wild-type from variant ATTR. While LF involvement is more commonly associated with ATTRwt, hereditary variants may also present with spinal manifestations (33). This will allow exploratory subgroup analyses comparing treatment response between ATTRwt and ATTRv populations and improve the biological characterization of the cohort. Additionally, baseline ligamentum flavum specimens will undergo semi-quantitative grading of amyloid burden (e.g., low vs. high deposition), as previously described in LF amyloid studies, with correlation to digital image-based quantification such as preoperative magnetic resonance imaging (MRI) scans in all lumbar levels (L1-S1) with machine learning analyses (34). Although repeat biopsies after tafamidis therapy may not be feasible, baseline quantification may provide insight into whether amyloid burden predicts treatment response.

There should be two arms, one using tafamidis standard dose for ATTR-CM (61 mg daily), and the other placebo, with a duration of 12–18 months, as LF thickening is a slow process that needs at least one year to detect radiological stabilization. Both arms should include 30–50 patients, sample size powered for signal detection rather than definitive efficacy. The primary endpoint would be the difference in progression of ligamentum flavum thickness at predefined lumbar levels on standardized, centrally reviewed MRI at 12 months. The secondary endpoints may be clinical (e.g., ODI, VAS back/leg pain, walking distance, reoperation rate at 24 months), radiological (spinal canal diameter and area change), and cardiac (echocardiographic strain, NT-proBNP elevation).

Recruitment feasibility is supported by the relatively high prevalence of ATTR deposition in the ligamentum flavum among patients undergoing surgery for lumbar spinal stenosis, reported in prior studies to range from 30% to 50% (10, 11). Based on these rates, a center performing ≥100 decompressive spine surgeries per year could potentially identify 30–50 eligible participants annually. A two-step screening strategy can be effectuated: a preoperative assessment, with a review of prior histological reports, patient age, and clinical risk factors (e.g., bilateral carpal tunnel syndrome, biceps tendon rupture) to identify likely candidates; and an intraoperative confirmation with tissue biopsy. Using this approach, we estimate that recruitment of the target cohort for a 12–18 month treatment arm is feasible within 12–24 months at high-volume spine centers, balancing rigorous patient selection with practical timelines.

In the Dominican Republic, real-world availability of tafamidis is extremely limited, with fewer than 10 patients currently receiving treatment, primarily due to cost and access barriers. This suggests that tafamidis use is uncommon across both early and advanced stages of ATTR, and the likelihood of inadvertent inclusion of pre-treated patients is low but not negligible. To address this potential confounder, the proposed study design will exclude patients receiving tafamidis at any disease stage prior to enrollment. Prior exposure will also be assessed during screening to ensure accurate classification. This approach is intended to improve attribution of observed effects on ligamentum flavum thickness and clinical outcomes to the intervention under investigation.

### 1.5 Economic and regulatory landscape

Tafamidis is an expensive drug, reaching 225k/year in some regions, which can limit accessibility by the majority of the population, even in developed countries (35). While this is true now, there is evidence of previous pharmacological treatments that initially were costly but are now affordable. For example, when the first direct antiviral agent (DAA) for hepatitis C (Sovaldi) initially cost 84k per a 12-week course, but newly added DAAs have reduced the cost to 23–25k per course, an almost 80% drop in just a decade (36). In addition, other drugs such as acoramidis, another TTR stabilizer, and vutrisiran, an RNA silencer, are showing promising results in treating ATTR-CM, and thus can be included in the treatment of LSS to increase competition (37). Also, generics are thought to be included in the market in the following years, a promising Vyndaqel biosimilar drug that has an expected entry date by 2029 by the latest, which can compete with Pfizer's Vyndaqel market shares (38). Regarding its use for ATTR-CM, it has been suggested that a 92.6% price reduction would be necessary to make tafamidis cost-effective (39). This may be a temporary hurdle rather than a permanent roadblock in accessibility.

Clinical trials are needed to clarify the benefits of tafamidis in LSS, in which drug penetration to ligamentous tissue and significant functional outcome recovery will be assessed. The repurposing of an already known drug to LSS, which affects 11% of the general population, mainly the elderly, can be of great benefit for both patients and pharmacological institutions (40).

## 2 Discussion

Based on the information disclosed, it is imperative to plan and conduct clinical trials that include both patients with ATTR and LSS as the primary objective. It remains possible that TTR deposition represents an age-associated epiphenomenon rather than a primary driver of ligament hypertrophy. In this context, amyloid deposition may act as a biomarker of tissue aging rather than a causative factor. However, the observed correlation between amyloid burden and ligament thickness suggests a potential contributory role, which warrants prospective investigation. A prospective pilot study using LF biopsy, measuring TTR deposits, and functional and radiological follow-up can adequately demonstrate any association between symptomatology improvement and the use of tafamidis in LSS patients. If validated, this approach could represent one of the first mechanism-targeted pharmacologic strategies directed at the biological substrate of degenerative LSS.
